# Retraction: The History and Mathematics of the N-Localizer for Stereotactic Neurosurgery

**DOI:** 10.7759/cureus.r4

**Published:** 2016-06-23

**Authors:** Russell A Brown, James A. Nelson

**Affiliations:** 1 Principal Engineer, Retired, Palo Alto, USA; 2 University of Washington School of Medicine

This article (Brown R A, Nelson J A. (January 16, 2014) The History and Mathematics of the N-Localizer for Stereotactic Neurosurgery. Cureus 6(1): e156. doi:10.7759/cureus.156) is being retracted because it has been replaced by updated and improved articles with additional information (http://www.cureus.com/articles/3144 Brown R A, Nelson J A (September 14, 2015) The Origin and History of the N-Localizer for Stereotactic Neurosurgery. Cureus 7(9): e323. doi:10.7759/cureus.323​), (http://www.cureus.com/articles/3162 Brown R A (October 02, 2015) The Mathematics of Three N-Localizers Used Together for Stereotactic Neurosurgery. Cureus 7(10): e341. doi:10.7759/cureus.341) and ("http://www.cureus.com/articles/4592 Brown R A, Nelson J A (June 14, 2016) The Invention and Early History of the N-Localizer for Stereotactic Neurosurgery. Cureus 8(6): e642. doi:10.7759/cureus.642).

